# $100,000 Premium

**Published:** 1883-12-20

**Authors:** 


### *$100,000 PREMIUM.*

At the American Health Association, which assembled last at Savannah, Georgia, a resolution was adopted to memorialize the Congress of the United States to offer a Premium of $100,000 for the "Discovery of the Cause of Yellow Fever, or any certain means of effecting its prevention, destruction or harmless modification."

